# Address Delivered before the Tennessee Dental Association, at Memphis, July 19th 1868, by the Retiring President

**Published:** 1869-03

**Authors:** W. H. Morgan


AMERICAN JOURNAL

OF

DENTAL SCIENCE.

Vol. 11. THIRD SERIES--MARCH, 1869. No. 11.

### ARTICLE I.

*Address delivered before the Tennessee Dental Association, at Memphis, July 19th 1868, by the retiring President.*

W. H. MORGAN, M. D., D. D. S.

GENTLEMEN :—Our Constitution requires an address at the hands of the outgoing President.

I appear before you to discharge that duty, and although I bring meager abilities to the performance of this task, I know you are prepared to make ample allowance for the deficiencies of one who this one time steps aside from the laborious duties of practical life.

One year ago this Association was formed and its purposes defined. I congratulate you upon the event, as the auspicious beginning of a new era of more extended usefulness in our chosen profession, a higher degree of dignity and a general promotion of all the interests of dentistry as an art and science.

I invoke your single and united effort in behalf of a purpose honorable to yourselves and your art. I regard this association as a bond of union between worthy and honorable members of our profession, intended for common advancement and protection, "giving the world assurance," of moral and intellectual fitness in our avocation and holding up to scorn the jugling arts of the mountebanks who have invaded with impiety the sacred temple of science.

Before proceeding farther it is my painful duty to announce the death of a youthful Brother, who did honor to himself and this association, by his energy and attainments.

It is thus that the lesson of our mortality is constantly enforced with solemn emphasis, and repeated demand made for gratitude, that we are spared for the prosecution of the great ends of our good, the general welfare of our race and the glory of God.

We are led to reflect that even the shortest life should not be in vain, since it presents opportunities for a continual growth in wisdom, virtue and power.

The life of every man with his talent and influence is but a part of the grand scheme of intellectual and spiritual existence, comprehending all nature and illustrating the perfection of the moral government of the universe. All occupy to each the relation of benificiaries and dependents, there can be no exemption of any individual from the imperative demand of his fellows upon his time, talents or other means committed to him for the general good. The moral law and the law of our nature is satisfied with nothing less than a persistent effort for the discharge of our whole duty, in individual and collective capacities. The great enterprises of society in some form or other enlist the intellect and labor of all its members according to capacity and inclination. Evangelization, commerce, government, charity, the mechanic and fine arts are some of the necessary subdivisions of the great aggregate of labor consequent upon our temporal and eternal welfare.

All labor is honorable and deserving reward in the degree of special results in its particular sphere. Our speciality is one of the humane and ameleorative arts which human suffering has called into life. If we consider the sad heritage of pain belonging to our nature, the anguish inseperable from our fallen state, restraining its victim from the active duties of life, the sympathetic prostration of mind consequent upon disease, in short if we consider the vast issues of life, we must regard the Physician and Dentist not less than the Clergyman and Philanthropist, as ministers to the highest good and happiness of the human race. Our art is one which directly preserves life and health, and therefore stands immediately related to all the benefit of a sound mind in a sound body. If then its object is noble and its pursuit invaluable in the sum of human good, our energy should be correspondingly active and efficient to enlarge the extent of its operations for the relief of human suffering.

As members of an honorable profession we owe duties to ourselves and society, which must be scrupulously observed, That we may demand without shame or misgiving the confidence of those to whom our services are indispensable. To each other we owe a generous sympathy and support, a consistent and firm respect growing out of devotion to a common end, the practice of manly virtue as the true claim to that respectability which of right belongs to the science of Dentistry. To society we owe industry, punctuality, and skill in all our dealings, and cheerful zeal in all its cherished objects; thus shall our profession be placed upon a sure foundation of esteem and we be strengthened in our laudable endeavor for usefulness. Our profession being liberal and scientific places its practitioners in a conspicuous position, marks for public criticism, for approbation or censure, according to the display or lack of honesty and capability.

The learning and skill of a D.D.S. should not merely secure his own profit and fame, but should be used to enhance the public estimation of the profession in its associated capacity, as being devoted to a science which commands the best mental and moral endowments and manual training as necessary requisites to proficiency and honor. Let every dentist love his profession, and with determination master it in spite of any fancied disadvantages under which he may suffer, and he will surely attain honor for himself and his calling. A man is unfit for any pursuit if he underrates its intrinsic importance, and the means of sure and permanent success A limited view of our art or science is a fatal barrier to progress and development, and no real attainments can be made without patient cultivation and toil, comprehensive of all special and collateral phenomena.

No science is eunique or independent, but all are so closely allied and interwoven that knowledge constitutes one grand whole of component but inseperable parts. I do not mean that proficiency in one branch of knowledge depends upon the attainment of all others, but that every science comprehends some of the principles and relations of others. Thus, geology is the structural history of the earth, and how vast the theme. How does the geologist account for the varying aspects of the earth's conformation? He must study chemistry and natural science to determine the character of heat and water, and their effects in the statification of the rocks, the deposit of soils, and the crystallization of the solids. And many other separate sciences forms part of this august one of geology. Chemistry by its laws of affinity, and methods of analysis, resolves all things into their component elements.

The domain of medicine is as extensive and vastly more important; our specialty is no exception to the general truth, and like the others, has no strict individuality. The claims of medicine in dentistry are now distinctly recognized as being a part of a thorough dental education.

Dentistry deals mainly with the teeth and gums, yet the various local and constitutional affections dependent upon organs require a knowledge of the whole system and the laws that govern it. Comparative anatomy presents an inexhaustible field to the dentist. A knowledge of special anatomy and physiology, their arrangement, and disposition of the functions of the body, the tissues, and their normal and abnormal conditions, are absolutely requisite to the intelligent practitioner of dentistry. Therapeutics as embracing Materia Medica and the practice of physic has also its claims to general acquaintanceship together with a knowledge of chemistry, both analytical and therapeutical.

Medical surgery and a good degree of mechanical skill are also necessary for obvious reasons which my time will not allow me to elaborate. The standard is high but we must reach it before we can legitimately claim the title of Drs. of Dental Surgery.

That knowledge is power has passed into a universal axiom, and, other things being equal as man's power is in proportion to his systematized knowledge. All of us have had occasion to use more than we possessed, and to regret the superficiality of our attainments in our noble science. The day is passed when dentists are regarded by the people as mere mouth carpenters and tooth joiners, whose only excellence is manual skill or sleight of hand. The earnest labors and large attainments of modern dentists, the published works, the transactions of societies and the high character of educated practitioners, have made their full impression upon general intelligence, and we now behold the supremely grand spectacle of a talented body of students and operators supported and recognized by a discriminating public.

This places us as individuals and as a body on high vantage ground, and we must see that nothing be lost which can be accomplished by liberal culture, by organization and by the exemplification of those principles so peculiary necessary to professional men.

I desire to call your attention to the necessity of organization as best adapted to the attaining of all to which I have alluded. Voluntary association is a marked characteristic of the present age. The bright acheivements in religion, politics and science are directly referable to union, acting as a focal centre, to which all separate rays emanating from every source are directed, the connection of mere numbers, of itself gives weight and respectabilty to an enterprise, add to this perfect concord of tastes and interests, a brotherly emulation to promote the common end, and we have a powerful advancement, not limited by any of the ordinary posibilities of human endeavor. In an association like this the man of science finds fit audience for his thought and disquisition, then he brings his offering which is aceepted with gladness, and here he reaps his reward and witnesses the triumph of his labor. Every member should labor with unceasing zeal to add somewhat to the general stock, confident that every sugestlon of truth may give birth to greater thought and enlarged view of a new discovery of truth. Nothing of value is lost but all research and applications of theory become historical and descend to posterity in the line of succession.

Our legitimate objects are the increase of technical and general knowledge relating to our art, a full preparation for the relief of suffering, and the promotion and elevation of our profession, in which it is our highest privilege to labor for wealth and fame.

Such associations are movements to dental science on which every member must lay his stone duly marked and polished, the public repositories of archives wherein is treasured up whatever is valuable in thought and available in practice. They are schools in which all distinctive titles are abolished so that the learner is at the same time teacher, and the teachers ready to learn from the novice what his own research has not discovered.

The history of Dental association in the U. S., is a pertinent proof of the value of organization, with small and dubious beginnings; they throw out annually the aggregate wisdom of such a mass of mind as to entitle them to take rank with the first institutions of the age for the propagation of science. Dentistry through their agency has attained a substantial progress, a maturity of members and influence surpassing the most sanguine expectation and furnishing the strongest incentive to continual exertion in this direction.

Let me invite your attention to local and sectional organization. The American Dental Association does not deserve its general appellation "national" as it is controlled by the interests of a section never having met on Southern soil since its organization at Washington in 1860.

There is a want of affiliation between the Northern and Southern elements of this association that should not exist between men of science united in a common brotherhood. Southern men enter into their councils with reluctance, this debars intimate personal relations in many instances, and is therefore unfriendly to the avowed object of the association.

If there be a single disturbing cause incompatible with honor on the part of either party, it is best that the relation cease, and that each be left to prosecute its designs undisturbed by the other. It is a matter,of deep regret that the grand object of scientific investigation does not banish any local and personal interest. Knowledge claims no section but demands the sacrifice of every prejudice inconsistent with its pure and exalted aim of good for all; like charity it is universal, capable of cementing a brotherhood of devotees, and of assimulating them to its own peaceful and harmonious spirit; this ought to be so, but the times are unfavorable.

It is painfully evident that the late war has denationalised many of onr interests and that the homogenious organizations of the *ante bellum* period must be succeeded by others that afford no matter for sectional rivalry and discussion. Aside from the reasons assigned above, other causes tend to give a sectional character to all organizations in this country; physical and climatic diversity, the various and opposite pursuits of the people, (determined by necessity or taste) produce local centres of trade, education and art. This condition of things is perhaps inevitable, if so we must suit our action thereto. If our grand object can be best secured by a Southern organization in which political and social congeniality will be an additional bond of brotherhood, let us act accordingly with proper speed and directness and thus give shape to our wishes. To my mind this course is inevitable. For similar reasons the South must educate her young men who are to enter our profession. The strife and bickering between Northern Dental Colleges tends to prevent real progress, besides sometimes subjecting individual students to mortification and insult revolting to self respect and conscious rectitude. Our necessities do not justify the patronage of Northern institutions, the ability and zeal of Southern D. D. S's. and M. D's. is fully equal to any demand of education upon them, I say it with pride.* The return of prosperity to the South will open a wide field to the dental profession, it is our duty if possible to see that none enter it but those who are worthy and well qualified. It will be well for the profession and the people if those who are to enter this field, to reap the profits and honor thereof are men of capacity high culture and honor.

To propose any plan for carrying out the foregoing sugestions, including the organization of southern schools and the development of southern literature, would be premature until we have secured a wide and active co-operation throughout the South. I insist however that it is high time for agitation and correspondence. To this whole subject, more briefly alluded to than its importance demands, I invite your serious consideration and discussion. Your attention is again called to the great importance of legislative enactment for the more efficient protection of the profession and general public, from the charlatans and empirics who threaten alike the honor of Dentistry and the health of their dupes. Quackery has been and continues to be the most dangerous foe to our art. The bold daring and unblushing pretending of the ignorant upstart, passes current for consumate skill and dexterity with those who are not sufficiently intelligent to unmask their hypocracy. Malpractice soon comes to light

---

* The writer would not be understood as intimating that Southern dentists have not contributed their full proportion to the advancement of our profession. *The American Journal of Dental Science,* [the first devoted to our profession] was issued simultaneously at Baltimore, Philadelphia, New York and Boston, June 1st, 1839, It soon after became a Baltimore periodical, and was for many years the only Journal devoted to our specialty. Professor H. H. Hayden of Baltimore, was the first President of the American Society of Dental Surgeons, and was called the "Father of American Dentistry," see Vol. 1st. Page 179, American Journal Dental Science. This Society was organized Aug. 18, 1840.

Maryland chartered the first Dental College, which was organized in 1839 The first State Society, [The Virginia Society of Surgeon Dentists,] was organized at Richmond. Va, Dec. 12th, 1842.

Alabama passed the first law recognizing us as a profession and regulating the practice of Dentistry, approved Dec. 3d, 1841

and the infamy belonging to the cheat is thrown upon the whole body of honorable practitioners. Dentistry is thus brought into contempt and robbed of its legitimate honors and rewards. The legislature of our state is slow to do us justice. Other states have salutary provisions placing dentistry on the same footing with medicine, requiring necessary qualifications and providing for board of examiners, this is just what we ask in this state, it is highly probable that Kentucky will grant the same measures through her next Legislature. Action upon this subject has already been taken by Ohio, Indiana, Illinois, Michigan, Pennsylvania and N. York; should these states pass stringent laws regulating the practice of Dentistry, then our unprotected States will be overrun by a migratory swam of hungry adventurers and charlatans, preying upon the easy faith of our people, robbing them of their money teeth, and health. Such bodies as this, through individual effort in the dissemination of general information, can do much to mitigate this threatened evil. But the law should prevent by penalty what we can only restrain by example, remonstrance and the education of the people.

---

